# PL-7 Antisynthetase Syndrome in Association with Sjögren's, Systemic Lupus Erythematosus, and Rheumatoid Arthritis

**DOI:** 10.1155/2020/4736476

**Published:** 2020-02-13

**Authors:** Mehrin Jawaid, Yael Ross, Mohammad Kamran

**Affiliations:** WellStar Kennestone Regional Medical Center, Marietta, GA 30060, USA

## Abstract

We present a rare case of PL-7 antisynthetase syndrome (ASS) in association with Sjögren's, systemic lupus erythematosus (SLE), and seropositive rheumatoid arthritis (RA). Initially, the patient was diagnosed with Sjögren's followed by Sjögren's/SLE overlap and then Sjögren's/SLE/RA overlap. She was eventually diagnosed with Sjögren's/SLE/RA overlap with PL-7 ASS with interstitial lung disease (ILD). ILD was discovered after complaints of pleuritic chest pain with subsequent workup with coronary computed tomography (CT) revealing pulmonary fibrosis. This case demonstrates the ambiguity with which symptoms of ASS can present; given the high respiratory morbidity and mortality of ASS especially in non-Jo-1 patients, those who present with Raynaud's, myositis, or joint pain, whether together or in isolation, should be assessed for presence of additional features of ASS and potentially undergo testing for ASS antibodies if appropriate.

## 1. Introduction

Antisynthetase syndrome (ASS) is a subgroup of idiopathic inflammatory myopathies (IIM) characterized by the presence of antisynthetase antibodies and at least one of the following: myositis, ILD, and inflammatory arthritis. Raynaud's, mechanic's hands, and fevers may be present as well. Given the lack of specific classification criteria, often ASS can be confused with arthritis or other connective tissue diseases. Various antisynthetase antibodies include anti-Jo-1, anti-PL-12, anti-PL-7, anti-OJ, anti-EJ, anti-KS, anti-Zo, and anti-YRS [1]. Prevalence of ASS remains unknown; however, IIM, which includes the ASS subset, has a prevalence of 10 per 100, 000 individuals [2]. Anti-Jo-1 is the most common ASS antibody with prevalence of 20–25% in IIM, and the remaining are very rare with prevalence between <1% and 5% [3].

## 2. Case Presentation

A 41-year-old African American female presented with complaints of fatigue, xerostomia, xerophthalmia, and mild polyarthralgias with less than ten minutes of morning stiffness. The patient denied fevers, unintentional weight loss, photosensitivity, alopecia, oral ulcers, serositis, blood clots, or unusual headaches. She had never been pregnant, was a life time nonsmoker, worked in an office setting, and did not take any medications. Family history was significant for rheumatoid arthritis in her father.

On physical exam, she exhibited dry oral mucosa. She did not exhibit any rashes, synovitis, or joint tenderness. Examination of all other organ systems was unremarkable. An antinuclear antibody by immunofluorescence was positive (1 : 160) with nucleolar pattern (ref >1 : 80 elevated). Double-stranded DNA (DsDNA), Smith, and ribonucleoprotein antibodies were negative. C3 was 89 mg/dL (ref 90–180). C4 was 10 mg/dL (ref 16–47). Sjögren's antibody (SSA) was 6.7 (ref <1), serum protein electrophoresis was normal, and rheumatoid factor negative. She was diagnosed with Sjögren's syndrome based on the symptoms of xerostomia and xerophthalmia, positive SSA antibody, and fatigue. Xerophthalmia was confirmed by her ophthalmologist via positive Schirmer test.

Approximately 18 months after her initial diagnosis, the patient developed photosensitivity with rash, headaches, increased hair loss, alopecia, and increased fatigue. She continued to experience xerostomia, xerophthalmia, and Raynaud's. Repeat serologies showed DsDNA antibodies were now positive at a titer of 1 : 10, (ref <1 : 10). Complements were low with C3 84 mg/dL and C4 8 mg/dL. Smith and ribonucleoprotein antibodies, complete blood count, complete metabolic panel, urinalysis, and antiphospholipid antibodies were normal. At that time, a diagnosis of Sjögren's/SLE overlap was made. She was started on hydroxychloroquine 200 mg twice daily.

She remained stable on hydroxychloroquine for over a year. On a follow-up visit, she complained of increased joint pain and swelling in her hands and knees associated with morning stiffness for more than two hours over the previous two months. On physical exam, she exhibited tenderness and synovitis of bilateral wrists, bilateral metacarpophalangeal, and bilateral proximal interphalangeal joints. Repeat serologies revealed rheumatoid factor (RF) of 167 IU/mL (ref < 14) and anticyclic citrullinated peptide antibody (CCP) > 250 (ref > 59 strong positive). Hand X-rays showed no erosions. The patient met the 2010 American College of Rheumatology criteria for the diagnosis of RA in addition to already diagnosed overlap of Sjögren's/SLE. Recommendation was made to start methotrexate, but she declined due to concern over side effect profile. She was unable to tolerate sulfasalazine and leflunomide due to symptoms of lightheadedness and hair loss, respectively, and was reluctant to initiate biologic agents due to concern over side effect profile. She managed her symptoms on hydroxychloroquine 200 mg twice daily and ibuprofen 200–400 mg three times daily as needed for pain. The patient did take a prednisone taper initially for 3 weeks and stopped thereafter.

**Figure 1:**
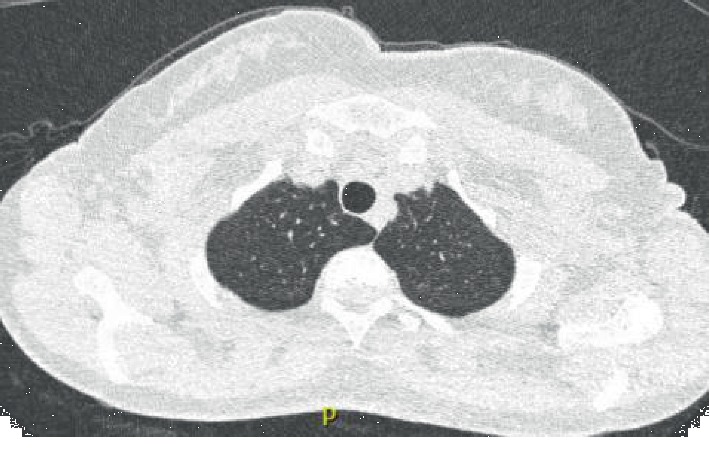
HRCT chest, prone image, and apical view.

**Figure 2:**
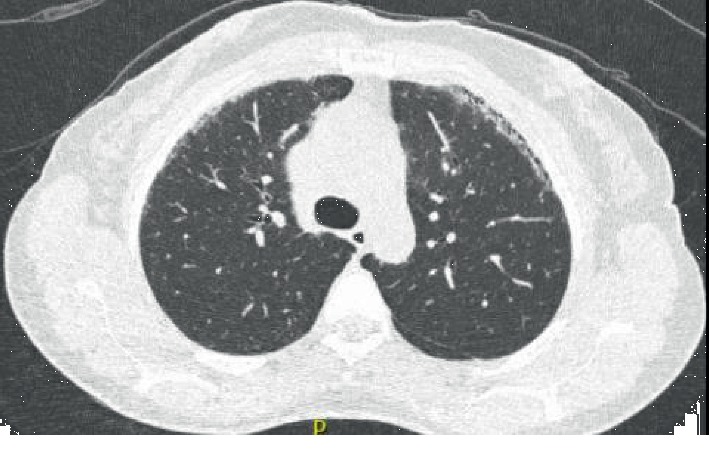
HRCT chest, prone image, and honeycombing in the anterior upper lobes.

**Figure 3:**
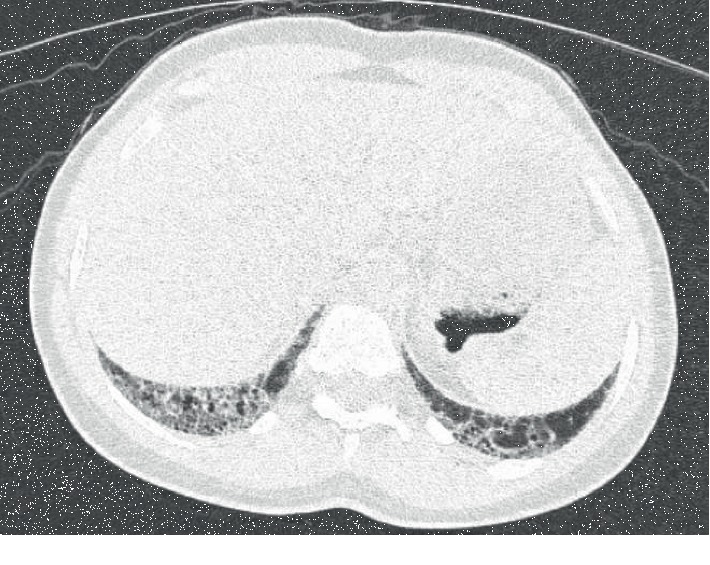
HRCT chest, prone image, honeycombing, and reticulation peripheral lung bases.

Due to complaints of pleuritic chest pain, the patient underwent workup which included a coronary CT scan, which revealed lung fibrosis. Follow-up high-resolution CT (HRCT) revealed multiple, noncalcified pulmonary nodules, the largest measuring 7 mm and mild honeycombing in the peripheries in a usual interstitial pneumonia (UIP) pattern (Figures 1–3). Given the findings on her CT scan, an ILD/myositis panel was ordered. Anti-PL-7 antibody was positive, 79 (ref < 11). An echocardiogram showed mild tricuspid regurgitation; pulmonary artery pressure was 26. Pulmonary function testing (PFT) was normal. A diagnosis of PL-7-positive antisynthetase syndrome with Sjögren's/SLE/RA overlap was made. Rituximab or mycophenolate was recommended; however, patient declined due to concern over side effect profile. On follow-up five months later, PFTs revealed moderately reduced diffusion lung capacity for carbon monoxide. She was evaluated at an ILD clinic at a university hospital where the diagnosis and treatment plan were agreed upon. After initial hesitation about starting medications, the patient agreed to start mycophenolate.

## 3. Discussion

This is an interesting rare case of overlap syndrome with PL-7 ASS, Sjögren's, systemic lupus erythematosus, and rheumatoid arthritis with evolving symptoms over time. ASS has often been reported in overlap with polymyositis, dermatomyositis, systemic sclerosis, Sjögren's, and RA [4–7].

Clinical heterogeneity is a feature of ASS with variable symptoms among different ASS antibody subgroups. Muscle involvement has been shown to be milder in patients with PL-7 antibodies compared to those with Jo-1 antibodies, including in those patients who have ASS in overlap with polymyositis/dermatomyositis [4, 5, 8]. There have also been reports of lower prevalence of mechanics hands and joint symptoms in PL-7 patients compared to Jo-1 patients [8, 9]. Pulmonary involvement can present as a spectrum from asymptomatic to progressive or acute lung disease. Pericardial disease, intestinal peudo-obstruction, and malignancy have been reported in ASS as well [9].

Interstitial lung disease is the largest contributor toward morbidity and mortality in ASS, especially in non-Jo-1 patients. It has been demonstrated that survival from time of diagnosis is significantly poorer in non-Jo-1 patients, and these patients also have increased pulmonary morbidity and mortality compared to Jo-1 positive patients [8]. In Marie et al.'s cohort of 15 anti-PL-7 patients, none of the patients had resolution of ILD. 35.7% of patients experienced deterioration of ILD with worsened functional status, and 7.1% died from lung complications [9]. Besides the presence of the non-Jo-1 antibody, concurrent presence of the Ro52 antibody, older age, and elevated serum ferritin have been associated with more rapidly progressive interstitial lung disease (RPILD) [10]. Those with the UIP pattern by HRCT have also demonstrated decreased steroid responsiveness and RPILD [9].

Nonspecific interstitial pneumonia (NSIP) and organizing pneumonia are the predominant HRCT patterns that have been reported in ASS [11, 12]; however, cases of ASS with UIP pattern have also been reported as with Marie et al.'s cohort of 15 patients of which 42.9% demonstrated UIP, 42.9% NSIP, and 14.2% with organizing pneumonia [9]. Yousem et al. also highlight the presence of the UIP pattern in biopsies of PL-7 patients [13].

Furthermore, the onset of ILD and initial presenting symptoms can vary in ASS. It has been shown in PL-7 patients that ILD could present either before, simultaneously, or after myositis presentation [9, 14]. Overall, in comparison to Jo-1 positive patients, non-Jo-1 patients tend to present with nonspecific connective tissue disease symptoms. There have also been cases where PL-7 patients have presented in overlap with various other diagnoses and as in our case, in overlap with Sjögren's, SLE, and RA. Thus, PL-7 ASS or other non-Jo-1 antibody syndromes could present at least initially, as idiopathic ILD, undifferentiated connective tissue disease, and/or evolve into or present as an overlap syndrome.

Our case demonstrates the ambiguity of initial presenting symptoms of non-Jo-1 patients, which is in line with the literature for presentation of most non-Jo-1 patients. Our patient initially presented with Raynaud's during her teenage years; then, in her 40s, she developed Sjögren's followed by Sjögren's/SLE overlap and then Sjögren's/SLE/RA overlap. She was eventually diagnosed with Sjögren's/SLE/RA in overlap with PL-7 ASS after complaints of pleuritic chest pain and CT revealing ILD with a positive ASS panel. The ambiguity of presenting symptoms is thought to contribute to delayed diagnosis and treatment of ASS which increases pulmonary morbidity and mortality, especially in non-Jo-1 patients, and contributes to poor survival [8]. When evaluating patients with connective tissue disease, careful attention should be paid to identifying features of ASS and potentially testing appropriate patients with ASS antibodies even in patients with rheumatoid arthritis with positive RF, CCP antibodies, and erosive changes on X-ray [15, 16].

Immunosuppressive agents treat manifestations of antisynthetase syndrome. Frequently used agents include azathioprine, mycophenolate mofetil, tacrolimus, rituximab, and cyclophosphamide, though there is little consensus on a single preferred age. The use of these agents to treat antisynthetase syndrome-related ILD is off-label [17]. In a recent 2018 retrospective review of 25 ASS patients treated with rituximab, in most patients, improvement or stability was demonstrated on chest CT and PFTs. Glucocorticoid requirements decreased, and the medication was well tolerated in most patients [18]. Rituximab was also found to be beneficial in ASS and seropositive RA overlap syndrome with joint efficacy and extra-articular improvement and or stability. On the contrary, in few patients, TNF alpha inhibitors resulted in exacerbation of myositis and respiratory status [7].

## 4. Learning Points

- ASS especially non-Jo-1 cases can present ambiguously as idiopathic ILD, undifferentiated connective tissue disease, and/or evolve into or present as an overlap syndrome; we present a unique case of PL-7 ASS in overlap with Sjögren's, RA, and SLE.
- Given the high respiratory morbidity and mortality of ASS patients, when evaluating patients with ILD, arthralgias, Raynaud's, or myositis, careful attention should be paid to identifying features of ASS, and the appropriate patients should be screened for ASS antibodies.
